# Impact of diabetes mellitus and glucose level control on early sepsis-associated acute kidney injury: a multicenter retrospective observational study

**DOI:** 10.3389/fmed.2026.1878791

**Published:** 2026-07-20

**Authors:** Lina Zhao, Qian Cui, Shuoyan Dong, Hongyan Wang, Fei Yang, Yunying Wang, Hui Shi, Xiaopeng Shi, Dongming Cao, Zhe Chen, Yongwang Wang, Yun Li

**Affiliations:** 1 Department of Critical Care Medicine, Tianjin Medical University General Hospital, Tianjin, China; 2 Department of Geriatrics, Tianjin Geriatrics Institute, Tianjin Medical University General Hospital, Tianjin, China; 3 Department of Respiratory and Critical Care Medicine, The First Affiliated Hospital of Baotou Medical College, Baotou, China; 4 Department of Respiratory and Critical Care Medicine, The Third Affiliated Hospital of Inner Mongolia Medical University, Baotou, China; 5 Department of Critical Care Medicine, Chifeng Municipal Hospital, Chifeng Clinical Medical College of Inner Mongolia Medical University, Chifeng, China; 6 Department of Emergency, Henan Provincial People’s Hospital, Zhengzhou University People’s Hospital, Zhengzhou, Henan, China; 7 Department of Intensive Care Unit, Liaocheng People’s Hospital, Liaocheng, Shandong, China; 8 Department of Anesthesiology, The Second Hospital of Tianjin Medical University, Tianjin, China

**Keywords:** acute kidney injury, diabetes mellitus, glucose level control, ICU, sepsis

## Abstract

**Purpose:**

Sepsis-associated acute kidney injury (SA-AKI) raises mortality risk, while the independent links of pre-existing diabetes mellitus (DM) and in-hospital glycemic status to SA-AKI remain unclear. This study explored their correlations with early SA-AKI among sepsis patients.

**Methods:**

This multicenter retrospective cohort enrolled 6,974 pathogen-positive adult sepsis patients admitted to ICUs from 2023 to 2026. We used pre-existing DM as the primary exposure and time-weighted average glucose as the secondary marker. Multiple adjustment methods including propensity score matching (PSM) and inverse probability weighting (IPW) were adopted to reduce confounding bias.

**Result:**

After enrolling 6,947 septic ICU patients, multivariate logistic regression identified pre-existing DM as an independent risk factor for early SA-AKI (adjusted OR = 2.54, 95% CI 2.25–2.86, *p* < 0.001). The protective glycemic range was 6–11 mmol/L for diabetic patients (adjusted OR = 0.88, 95% CI 0.78–0.98, *p* = 0.025) and 8–13 mmol/L for non-diabetic patients (adjusted OR = 0.55, 95% CI 0.48–0.62, *p* < 0.001). PSM, IPW and doubly robust estimation all validated these consistent associations. Abnormal glucose showed weak predictive power for SA-AKI (AUC 0.60-0.61). Higher time-weighted glucose correlated with advanced AKI stages and increased in-hospital mortality in mild-to-moderate SA-AKI patients. Diabetic SA-AKI patients had higher proportions of stage 1 and stage 3 AKI without significant difference in overall in-hospital mortality compared with non-diabetic counterparts. Matched SA-AKI patients suffered longer ICU/hospital stays, more organ support use and higher hospital mortality.

**Conclusion:**

Common pathogenic organisms had higher detection rates in SA-AKI cases. Pre-existing DM was positively correlated with early SA-AKI. Targeted in-hospital glycemic intervals were linked to lower SA-AKI risk, which supports individualized glucose monitoring for sepsis patients.

## Introduction

SA-AKI represents a life-threatening sepsis complication and drives high morbidity and mortality in critically ill populations (1). Around 48.9 million sepsis cases occur worldwide each year, and half of these patients may develop SA-AKI with a 20% mortality risk (2). SA-AKI arises from overlapping inflammation, microvascular damage and metabolic disorder, which jointly cause renal hypoperfusion and tubular damage (3). Despite significant advancements in sepsis management, SA-AKI remains a major clinical challenge due to the limited availability of effective therapeutic options to prevent or halt its progression.

Emerging evidence indicates that pre-existing diabetes mellitus (DM) may exacerbate the risk of SA-AKI through mechanisms such as chronic inflammation, endothelial dysfunction, and dysregulated immune responses (4). Hyperglycemia, a common feature in both diabetic and non-diabetic sepsis patients, has been linked to worsened renal outcomes by exacerbating oxidative stress and amplifying inflammatory cascades (5, 6). However, the relationship between glucose level control and SA-AKI risk remains poorly understood, with conflicting evidence regarding optimal glucose targets for different patient populations.

The glucose level control in sepsis patients presents both opportunities and challenges (7). While intensive glucose management may reduce the incidence of SA-AKI, it carries the risk of hypoglycemia, which can further compromise organ function. Recent studies have emphasized the potential benefits of individualized oxygen and blood pressure targets (8, 9). No prior research has identified suitable glycemic thresholds for patients complicated by both sepsis and AKI (7). Clinical glucose management is hard to standardize because individual metabolic responses differ widely.

This study fills the above research gaps by analyzing correlations between pre-existing DM, in-hospital glucose levels and early SA-AKI, and identifying group-specific glycemic ranges associated with lower SA-AKI odds.

## Methods

### Study design and patient population

This multicenter retrospective observational study was conducted across three hospitals from January 2023 to March 2026. Data collection periods across participating institutions were as follows: The First Affiliated Hospital of Baotou Medical College, Liaocheng People’s Hospital, and Chifeng Municipal Hospital, including all hospitalized patients who met the Sepsis-3.0 diagnostic criteria. Inclusion criteria were age >18 years, an expected hospital stay ≥48 h, and availability of follow-up data until hospital discharge or death. Patients with pre-existing chronic kidney disease (CKD) stages or missing blood glucose values were excluded. To ensure patient privacy, all data were anonymized. The study protocol was approved by the institutional review boards (IRBs) of all participating hospitals. As an observational study, the requirement for written informed consent was waived by the IRBs in accordance with the principles of the Declaration of Helsinki. The study adhered to the Strengthening the Reporting of Observational Studies in Epidemiology (STROBE) guidelines to ensure transparent and accurate reporting. No artificial intelligence software was applied in this study.

### Standard ICU care and glucose monitoring protocol

All enrolled sepsis patients received standardized ICU supportive care consistent with international Surviving Sepsis Campaign guidelines (2021 edition), including early broad-spectrum antimicrobial administration, fluid resuscitation, vasopressor support for hypotension, mechanical ventilation when indicated, electrolyte correction, and renal protective medication stewardship to minimize avoidable nephrotoxin exposure. Standard monitoring rules were applied uniformly in all three ICUs for sepsis patients. Continuous non-invasive blood pressure monitoring and hourly urine output recording were required. Blood gas, lactate and glucose tests were collected every 4 hours via arterial or peripheral venous access.

### Blood glucose monitoring workflow and observation window

Venous blood glucose samples were collected via peripheral venipuncture or indwelling central venous catheters every 4 h starting immediately upon ICU admission following sepsis diagnosis, covering the full 48 h pre-outcome observation window for early SA-AKI diagnosis. Point-of-care bedside glucose analyzers calibrated daily per hospital laboratory standards were used for all measurements; all raw glucose values were automatically archived into the centralized EMR system. The primary glucose exposure metric for analysis was the time-weighted mean glucose concentration calculated across all serial 4-hourly measurements within the 0–48 h ICU admission window prior to early SA-AKI adjudication. Glucose monitoring was continued beyond 48 h for secondary outcome analysis of in-hospital mortality, but only the 0–48 h serial values were used to derive the primary exposure time-weighted mean glucose for SA-AKI risk modeling.

### Data collection procedure and data anonymization protocol

A standardized multi-step data extraction procedure was implemented uniformly across all three participating hospitals to eliminate inter-center collection bias:

#### EMR screening

Trained clinical research coordinators at each institution ran pre-programmed structured query language (SQL) filters on the hospital EMR database to identify all patients with Sepsis-3.0 ICD-10 diagnostic codes admitted between January 2023 and March 2026.

#### Variable extraction

Predefined electronic case report forms (eCRFs) were used to extract baseline demographic data, pre-admission comorbidity histories, serial laboratory results, microbial culture data, ICU severity scores, medication administration records, glucose monitoring logs, fluid balance, urine output, organ support therapy, and discharge survival status.

#### Data quality control

Two independent research coordinators cross-checked 10% randomly sampled patient records at each center to resolve inconsistent, outlier, or missing laboratory values; discrepancies were adjudicated by a senior critical care physician blinded to study outcomes.

#### Centralized data merging

De-identified eCRF datasets from each hospital were encrypted and transferred to a unified central research database hosted, with all institutional site markers retained as a covariate for adjustment in multivariable models.

#### Missing data classification

Missing blood glucose values were defined as ≥3 consecutive unrecorded 4-hourly measurements within the 48 h window; patients meeting this threshold were excluded from the final cohort, while sporadic single missing glucose readings within the window were retained and handled via multiple imputation during statistical analysis.

### Definitions and outcome measures

Early SA-AKI was defined in accordance with the consensus report of the Acute Disease Quality Initiative (ADQI), based on the presence of sepsis criteria (as defined by Sepsis-3.0) and AKI criteria (as defined by the Kidney Disease: Improving Global Outcomes [KDIGO] guidelines) within 48 h of ICU admission (3, 10, 11). Late SA-AKI, defined as cases occurring between 48 h and 7 days after sepsis diagnosis, was excluded from the analysis due to the unavailability of serum creatinine data beyond 48 h. The primary outcome was the incidence of early SA-AKI stages 1 to 3, with baseline creatinine levels determined as the serum creatinine concentration at the time of sepsis diagnosis. AKI was defined using serum creatinine criteria. The secondary outcome was in-hospital mortality. Blood glucose levels were monitored every 4 h during hospitalization, and the average values were used for analysis.

### Critical temporal sequence of key variables

1. Pre-existing DM (primary exposure): Confirmed via historical medical records as diabetes diagnosed before ICU admission and sepsis onset; this variable is fixed at baseline and temporally precedes all in-hospital clinical events.
2. Early in-hospital glucose monitoring & critical illness scoring (secondary exposure/covariates): Serial blood glucose was sampled every 4 h starting immediately upon ICU admission post-sepsis diagnosis; SOFA, APACHE II, and GCS scores were calculated within the first 24 h of ICU admission. All glucose and severity data were captured during the 48 h pre-AKI observation window, prior to the time point used to diagnose early SA-AKI.
3. Primary outcome (early SA-AKI): Diagnosed if KDIGO AKI criteria were met within 48 h of ICU admission (terminal endpoint occurring after baseline DM status and early glycemic exposure).
4. Secondary outcome (in-hospital mortality): Recorded at hospital discharge or patient death, occurring subsequent to early SA-AKI diagnosis.

### Ascertainment criteria for pre-existing diabetes mellitus

Pre-existing DM was definitively ascertained via a multi-source verification protocol combining four complementary clinical data streams from pre-admission and admission EMR records, with positive confirmation from ≥1 of the following criteria required for DM classification:

#### Historical outpatient medical history

Formal physician-documented type 1 or type 2 diabetes mellitus diagnosis in primary care or specialist endocrinology records prior to ICU admission.

#### Chronic hypoglycemic medication exposure

Continuous pre-hospital prescription records of insulin, metformin, sodium-glucose cotransporter 2 inhibitors, sulfonylureas, or other oral anti-diabetic agents documented in outpatient pharmacy refill logs.

#### Admission glycated hemoglobin (HbA1c)

HbA1c laboratory testing performed within 7 days before or on ICU admission with result ≥6.5%.

#### Admission random venous glucose

Two separate random admission glucose measurements ≥11.1 mmol/L paired with classic hyperglycemic clinical symptoms, in the absence of acute stress-induced transient hyperglycemia without prior diabetic history.

Patients meeting none of the above four criteria were classified as non-DM sepsis patients for stratified glucose target analysis. Stress hyperglycemia occurring exclusively after ICU admission without any pre-admission diabetic evidence was categorized into the non-DM subgroup (12).

### Blood glucose monitoring workflow and observation window

To eliminate confounding from post-AKI glycemic measurements, the time-weighted mean glucose used for primary SA-AKI risk analysis was strictly restricted to all valid 4-hourly glucose samples collected within the first 48 h following ICU admission, prior to early SA-AKI diagnosis. No glucose data captured after the 48 h cutoff or after AKI onset was included in primary regression, PSM, IPW, or doubly robust models; post-48 h glucose values were only analyzed for the secondary outcome of in-hospital mortality. For participants who experienced early death or hospital discharge before the full 48 h monitoring window, their time-weighted mean glucose was calculated using only serial glucose results recorded up to their exact exit timepoint, without artificial filling of unmeasured time intervals after patient loss to follow-up. Per our pre-specified exclusion criteria, subjects with three or more consecutive missing glucose records within their available ICU stay were excluded entirely; sporadic single missing glucose readings were processed via multiple imputation for aggregate glucose estimation.

### Statistical analysis

All statistical analyses were performed using R statistical software (version 4.2.1). All continuous clinical indices were assessed for normality via Kolmogorov–Smirnov tests; nearly all laboratory and vital sign indicators exhibited non-normal distributions and were summarized using interquartile ranges (IQRs), while categorical variables were reported as absolute counts and column percentages. For between-group comparisons of continuous variables, Mann–Whitney *U* test was adopted for pairwise comparison (SA-AKI vs. non-SA-AKI), and Kruskal-Wallis *H* test was only used for comparisons across three or more independent subgroups. For categorical variables, Pearson *χ*^2^ test was applied when all cell expected counts ≥5; Fisher’s exact test was utilized if any cell expected count was less than 5. Kaplan–Meier (KM) survival curves were constructed to compare in-hospital survival between DM and non-DM SA-AKI subgroups, with log-rank test for inter-group survival difference testing.

Derivation of glucose concentration cut-off thresholds: Generalized additive models (GAM) with smooth spline functions were first applied to explore the continuous nonlinear dose–response relationship between time-weighted mean glucose and incident early SA-AKI, stratified by DM status. The *y*-axis of GAM plots adopts log-odds scale by default for binary outcome regression. Visual inflection points on the GAM risk curves were identified to mark glucose ranges associated with minimal SA-AKI odds for DM and non-DM subgroups separately. Subsequent stratified threshold sensitivity and specificity screening confirmed the optimal protective glucose bands: 6–11 mmol/L for patients with pre-existing DM, and 8–13 mmol/L for patients without DM. These two glucose target intervals were retained as the primary exposure strata for all multivariable adjusted risk models. We sequentially adopted multivariable logistic regression, 1:1 nearest-neighbor propensity score matching (PSM), inverse probability weighting (IPW), and doubly robust estimation to quantify the independent associations between pre-existing diabetes mellitus (DM), in-hospital glycemic control within predefined target ranges, and the incidence of early stress-associated acute kidney injury (SA-AKI); for our multivariable logistic models, all adjusted covariates, including demographics, comorbidities, infection characteristics, pathogenic species, vital signs, laboratory indicators, clinical interventions and study center, have been fully listed in Supplementary Tables 2–4, and we performed variance inflation factor (VIF) analysis to assess multicollinearity, confirming that all covariates retained in the final models presented VIF values below 5 with no evident collinearity, while PSM was implemented with a matching caliper set to 0.1 times the standard deviation of the logit-transformed propensity score, and covariate balance was assessed using absolute standardized mean difference (SMD), whereby an SMD < 0.1 was regarded as adequate balance, with corresponding balance results presented in Supplementary Figure 2, stabilized IPW was conducted as a dedicated sensitivity analysis to validate the robustness of findings derived from primary regression and PSM analyses, and doubly robust estimation was further applied to mitigate residual confounding, prior to all modeling procedures, laboratory outliers falling outside the 3 × interquartile range were winsorized at the 1st and 99th percentiles, and we adopted specific strategies to handle missing data: patients with three or more consecutive missing 4-hourly glucose measurements within the 48 h observation window were excluded from the study cohort, while isolated sporadic missing laboratory values were addressed via multiple imputation by chained equations (MICE), generating 20 imputed datasets whose effect sizes were pooled using Rubin’s rules for summary estimates reported in the Supplementary material, and all regression models were additionally adjusted for age, sex, mean arterial pressure, distribution of infection sites, categories of pathogens, administration of nephrotoxic antibiotics, baseline lactate, hemoglobin, platelet count, and participating hospital study site. All statistical analyses were performed using R statistical software (version 4.2.1), with a two-sided *p-*value <0.05 defined as the threshold for statistical significance.

## Results

### Study participants and baseline characteristics

A total of 10,029 critically ill patients diagnosed with sepsis were screened for eligibility between January 2023 and March 2026. A total of 1,368 individuals with pre-existing chronic kidney disease, 456 patients with incomplete serial blood glucose measurements within the 48-h observation window, and 1,258 patients with an ICU stay shorter than 48 h were excluded from analysis, leaving a final study population of 6,947 patients. The full cohort was stratified into patients complicated with early sepsis-associated acute kidney injury and those without SA-AKI, with all subgroup sample sizes clearly annotated within Table 1 and Supplementary material.

**Table 1:** Baseline characteristics of sepsis AKI patients.

	Original cohort			Match cohort		
	Non-SA-AKI patients (*n* = 3,685)	SA-AKI patients (*n* = 3,262)	*p*	Non-SA-AKI patients (*n* = 2,248)	SA-AKI patients (*n* = 2,248)	*p*
Baseline variables						
Age (years) (median [IQR])	69.00 [60.00, 77.00]	71.00 [61.00, 80.00]	<0.001	70.00 [61.00, 78.00]	70.00 [60.00, 79.00]	0.356
Gender, M (%)	1,275 (34.6)	1,259 (38.6)	0.001	824 (36.7)	822 (36.6)	0.975
Coexisting illness, (*n* (%))						
Hypertension	1,319 (35.8)	966 (29.6)	0.003	503 (22.4)	652 (29.0)	<0.001
Diabetes	1,174 (31.9)	1,830 (56.1)	<0.001	800 (35.6)	1,215 (54.0)	<0.001
Chronic obstructive pulmonary disease	724 (19.6)	822 (25.2)	<0.001	465 (20.7)	542 (24.1)	0.007
Coronary atherosclerotic heart disease	1,447 (39.3)	1,655 (50.7)	<0.001	878 (39.1)	1,174 (52.2)	<0.001
Site of infection, (*n* (%))						
Urinary	188 (5.1)	407 (12.5)	<0.001	162 (7.2)	167 (7.4)	0.819
Lung	139 (3.8)	389 (11.9)	<0.001	127 (5.6)	137 (6.1)	0.568
Catheter	61 (1.7)	153 (4.7)	<0.001	54 (2.4)	59 (2.6)	0.703
Skin and soft tissue	74 (2.0)	246 (7.5)	<0.001	70 (3.1)	71 (3.2)	1.000
Abdominal	62 (1.7)	230 (7.1)	<0.001	59 (2.6)	59 (2.6)	1.000
Kidney function (median [IQR])						
Creatinine (mg/dL)	0.90 [0.80, 1.20]	2.60 [1.70, 3.40]	<0.001	1.12 [0.85, 1.45]	1.26 [0.93, 1.58]	0.540
Blood urea nitrogen (mg/dL)	17.00 [13.00, 21.00]	29.00 [20.00, 43.00]	<0.001	17.00 [13.00, 22.00]	29.00 [20.00, 43.00]	<0.001
AKI staging, (*n* (%))						
1	0 (0.0)	1,573 (48.2)	<0.001	0 (0.0)	998 (44.4)	<0.001
2	0 (0.0)	1,357 (41.6)		0 (0.0)	974 (43.3)	
3	0 (0.0)	332 (10.2)		0 (0.0)	276 (12.3)	
Medication and biochemical index						
Nephrotoxic antimicrobial drugs, (*n* (%))	1,587 (43.1)	1,555 (47.7)	<0.001	1,036 (46.1)	1,012 (45.0)	0.491
Glucose (mmol/L)	11.22 [8.00, 12.22]	11.56 [9.00, 13.78]	<0.001	11.17 [8.06, 12.22]	12.39 [9.48, 14.94]	<0.001

AKI, acute kidney injury; IQR, interquartile range; SA, sepsis-associated. *p* < 0.05 was considered statistically significant.

Statistically significant intergroup disparities across all baseline clinical, laboratory and microbiological variables were observed (all *p* < 0.001). The median age of patients with SA-AKI was 71 years (interquartile range [IQR], 60–80 years), slightly higher than the median age of 69 years (IQR, 60–77 years) recorded in patients without SA-AKI. Pre-existing DM was substantially more prevalent among SA-AKI patients, affecting 56.1% (1,204/2,146) of this subgroup versus only 31.5% (1,521/4,828) of patients free from early AKI (Table 1).

Patients with SA-AKI demonstrated more severe physiological derangements upon ICU admission compared with non-AKI counterparts, including lower mean arterial pressure, elevated circulating lactate concentrations, reduced hemoglobin and platelet counts, higher serum creatinine and blood urea nitrogen, and diminished daily urine output. Exposure to nephrotoxic antimicrobial agents was also more frequent in the SA-AKI cohort (47.7%) compared with patients without SA-AKI (43.1%; *p* < 0.001). The median time-weighted mean blood glucose measured across the initial 48 h of ICU admission was moderately higher in patients who subsequently developed SA-AKI relative to those without AKI. We observed significantly higher culture positivity of *Escherichia coli*, *Staphylococcus aureus*, *Klebsiella pneumoniae* and *Pseudomonas aeruginosa* among patients with early SA-AKI (21.1, 34.7, 12.7, 8.1%) relative to non-AKI sepsis patients (7.5, 20.5, 3.2, 1.9%, all *p* < 0.001). Importantly, pathogen-positive sepsis was a fixed enrollment standard for this cohort, meaning all bacterial infections preceded early SA-AKI diagnosis. The elevated pathogen detection frequency in the AKI subgroup likely stems from aggravated systemic inflammation and more critical illness burden rather than representing a secondary complication induced by AKI (Table 1, Supplementary Table 1).

After matching, the prevalence of pre-existing DM remained significantly higher in patients with SA-AKI (51.3% versus 33.5%), and the median time-weighted blood glucose concentration was substantially elevated within the AKI subgroup (14.17 mmol/L versus 11.22 mmol/L). Covariate balance following matching was confirmed via absolute standardized mean differences below 0.1 for all adjusted variables, as detailed in Supplementary Figure 2. Consistent with observations from the full unadjusted cohort, matched SA-AKI patients exhibited higher Sequential Organ Failure Assessment (SOFA) and Acute Physiology and Chronic Health Evaluation II (APACHE II) scores, lower Glasgow Coma Scale (GCS) scores, greater reliance on continuous renal replacement therapy, prolonged ICU and hospital length of stay, and higher all-cause in-hospital mortality, as summarized in Table 2.

**Table 2:** The outcome of sepsis AKI patients.

	Original cohort			Match cohort		
	Non-SA-AKI patients (*n* = 3,685)	SA-AKI patients (*n* = 3,262)	*P*	Non-SA-AKI patients (*n* = 2,248)	SA-AKI patients (*n* = 2,248)	*P*
The score system, (median [IQR])						
SOFA	4.00 [3.00, 6.00]	6.00 [4.00, 9.00]	<0.001	4.00 [3.00, 6.00]	6.00 [4.00, 9.00]	<0.001
Non-AKI-SOFA	4.00 [3.00, 6.00]	5.00 [3.00, 8.00]	<0.001	4.00 [3.00, 6.00]	5.00 [3.00, 8.00]	<0.001
APACHE II	18.00 [12.00, 22.00]	21.00 [16.00, 23.75]	<0.001	17.00 [12.00, 22.00]	22.00 [18.00, 25.00]	<0.001
GCS	14.00 [11.00, 15.00]	14.00 [9.00, 15.00]	<0.001	14.00 [11.00, 15.00]	14.00 [9.00, 15.00]	<0.001
Treatment measures, *n* (%)						
Use of vasopressors	2,670 (72.4)	2,176 (66.0)	<0.001	1,535 (68.3)	1,546 (68.8)	0.748
Mechanical ventilation	3,322 (90.1)	2,534 (76.8)	<0.001	2,003 (89.1)	1,808 (80.4)	<0.001
CRRT	72 (2.0)	274 (8.3)	<0.001	53 (2.4)	199 (8.9)	<0.001
Hospital stay and prognosis						
Length of hospital stays, days (median [IQR])	10.70 [9.10, 14.00]	15.60 [10.70, 24.25]	<0.001	7.00 [5.20, 10.90]	11.60 [6.80, 19.90]	<0.001
Length of ICU stays, days (median [IQR])	6.08 [5.25, 7.42]	7.22 [5.72, 11.25]	<0.001	2.15 [1.26, 3.77]	3.45 [1.82, 7.74]	<0.001
Hospital mortality, (*n* (%))	190 (5.2)	803 (24.3)	<0.001	131 (5.8)	644 (28.6)	<0.001

AKI, acute kidney injury; APACHE II, Acute Physiology and Chronic Health Evaluation II; CRRT, continuous renal replacement therapy; GCS, Glasgow Coma Scale; IQR, interquartile range; SA, sepsis-associated; SOFA, Sequential Organ Failure Assessment. *p* < 0.05 was considered statistically significant.

### Primary outcome: independent associations between pre-existing DM, glycaemic control and early SA-AKI risk

Multiple complementary statistical frameworks were applied to quantify the independent association between pre-existing DM and incident early SA-AKI, including multivariable logistic regression, propensity score matching, inverse probability weighting and doubly robust estimation, all of which yielded consistent evidence that pre-existing DM represented an independent risk factor for early SA-AKI onset. After comprehensive adjustment for demographic features, comorbidities, infection characteristics, pathogen distribution, laboratory parameters and study center. Multivariable logistic regression demonstrated that pre-existing diabetes mellitus was associated with significantly elevated odds of developing SA-AKI, with an adjusted odds ratio (OR) of 2.54 and corresponding 95% confidence interval (CI) spanning 2.25 to 2.86 (*p* < 0.001). Analysis within the propensity score-matched cohort returned an OR of 2.22 (95% CI 1.97–2.50, *p* < 0.001), while inverse probability weighting (IPW) produced an OR of 2.18 (95% CI 1.96–2.43, *p* < 0.001); doubly robust estimation incorporating all covariates further validated this positive association with an OR of 1.44 (95% CI 1.37–1.50, *p* < 0.001).

**Figure 1:**
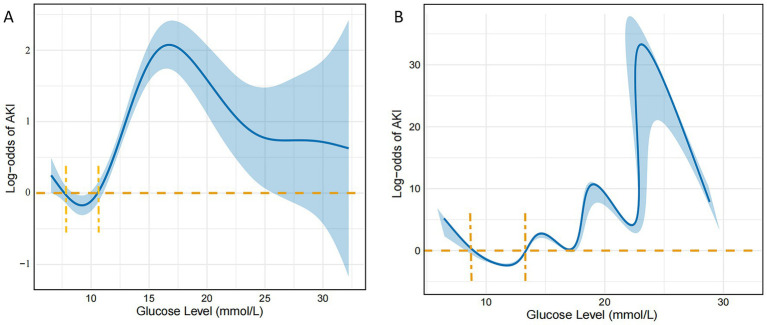
Nonlinear log-odds association between time-weighted mean blood glucose and incident early SA-AKI. The vertical axis of GAM curves is log-odds of SA-AKI risk, the standard output scale for binary regression models; Supplementary Figure 5 displays identical curves on absolute 0-1 risk probability scale for easier clinical reading. **(A)** Curve for patients with pre-existing diabetes mellitus, optimal protective glucose range = 6–11 mmol/L; **(B)** Curve for patients without diabetes mellitus, optimal protective glucose range = 8–13 mmol/L.

Generalized additive models with smooth spline functions were applied to characterize the non-linear dose–response relationship between blood glucose levels and SA-AKI risk stratified by diabetic status, with inflection points on risk curves identifying protective glycaemic target ranges (Figure 1). Among sepsis patients with pre-existing diabetes mellitus, blood glucose maintained at 6–11 mmol/L was consistently linked to reduced SA-AKI risk across all four analytical models. The adjusted OR was 0.88 (95% CI 0.78–0.98, *p* = 0.025) via multivariable logistic regression, 0.55 (95% CI 0.49–0.62, *p* < 0.001) via propensity score matching (PSM), 0.84 (95% CI 0.76–0.94, *p* = 0.002) via IPW, and 0.94 (95% CI 0.89–0.98, *p* < 0.001) via doubly robust regression. For patients without pre-existing diabetes mellitus, the protective glycaemic threshold was 8–13 mmol/L, which also consistently mitigated SA-AKI risk in all models. Multivariable logistic adjustment delivered an OR of 0.55 (95% CI 0.48–0.62, *p* < 0.001), propensity score-matched analysis returned an OR of 0.43 (95% CI 0.38–0.48, *p* < 0.001), IPW yielded an OR of 0.54 (95% CI 0.48–0.60, *p* < 0.001), and doubly robust estimation produced an OR of 0.76 (95% CI 0.73–0.80, *p* < 0.001). Complete stratified regression results are summarized in Table 3, Supplementary Tables 2–4.

**Table 3:** Multiple models assessed the relationship between diabetes mellitus and blood glucose levels and the incidence of AKI in sepsis.

Models	OR	95% CI		*p*
		2.5%	97.5%	
Multivariate logistic analysis				
Pre-existing diabetes mellitus	2.54	2.25	2.86	<0.001
Blood glucose level 6–11 (mmol/L)^*^	0.88	0.78	0.98	0.025
Blood glucose level 8–13 (mmol/L)^#^	0.55	0.48	0.62	<0.001
Propensity score matching				
Pre-existing diabetes mellitus	2.22	1.97	2.50	<0.001
Blood glucose level 6–11 (mmol/L)^*^	0.55	0.49	0.62	<0.001
Blood glucose level 8–13 (mmol/L)^#^	0.43	0.38	0.48	<0.001
Propensity score IPW				
Pre-existing diabetes mellitus	2.18	1.96	2.43	<0.001
Blood glucose level 6–11 (mmol/L)^*^	0.84	0.76	0.94	0.002
Blood glucose level 8–13 (mmol/L)^#^	0.54	0.48	0.60	<0.001
Doubly robust with all covariates				
Pre-existing diabetes mellitus	1.44	1.37	1.50	<0.001
Blood glucose level 6–11 (mmol/L)^*^	0.94	0.89	0.98	<0.001
Blood glucose level 8–13 (mmol/L)^#^	0.76	0.73	0.80	<0.001

IPW, inverse probability weighting; ^*^Patients with pre-existing mellitus; ^#^Patients without pre-existing mellitus; *p* < 0.05 was considered statistically significant.

Receiver operating characteristic curve analysis was performed to evaluate the predictive performance of out-of-target glucose ranges for early SA-AKI diagnosis (Supplementary Figure 3). For patients with pre-existing DM, failure to maintain blood glucose within the 6–11 mmol/L range demonstrated limited diagnostic utility for SA-AKI, with an area under the curve of 0.60, sensitivity of 0.59 and specificity of 0.60. Comparable modest predictive efficacy was observed in non-DM patients with glucose levels outside the 8–13 mmol/L target, with an area under the curve of 0.61, sensitivity of 0.76 and specificity of 0.47.

### Secondary outcomes: SA-AKI severity and in-hospital mortality

**Figure 2:**
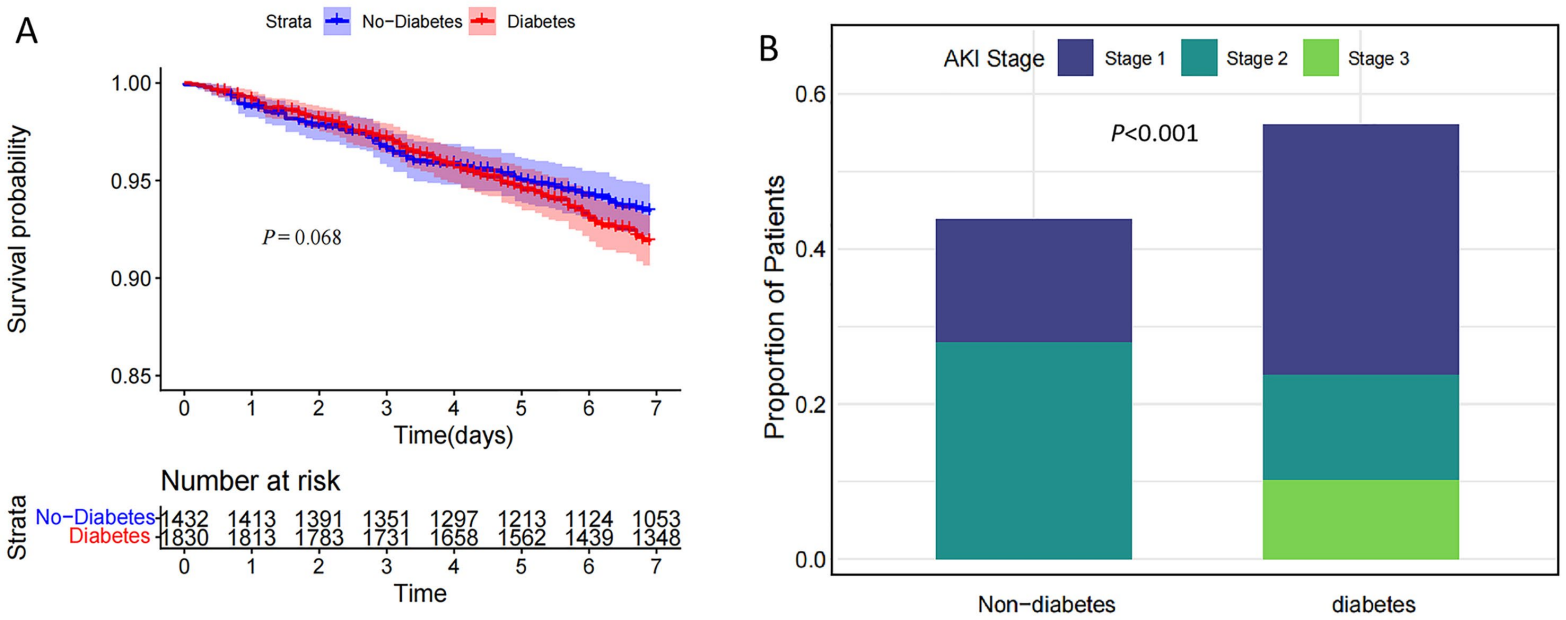
The relationship between diabetes mellitus and the prognosis and severity of AKI in patients with sepsis. **(A)** Kaplan–Meier curves of in-hospital mortality in patients with diabetes mellitus versus AKI with sepsis (*p* > 0.05). **(B)** The relationship between diabetes mellitus and the severity of AKI in patients with sepsis (*p* < 0.001).

Kaplan–Meier survival analysis with log-rank testing was conducted to compare hospital stay in the past 7 days trajectories between DM and non-DM patients complicated by SA-AKI, with no statistically significant difference in overall survival detected across groups (*p* > 0.05, Figure 2A). However, stratification by AKI stage revealed that patients with pre-existing DM experienced significantly higher rates of both stage 1 and stage 3 early SA-AKI relative to individuals without diabetes (*p* < 0.01, Figure 2B). Elevated in-hospital time-weighted mean glucose concentrations were significantly correlated with more advanced KDIGO AKI staging (Supplementary Figure 4A). Subgroup analysis restricted to patients with stage 1 and stage 2 SA-AKI further demonstrated that higher glycaemic levels were associated with increased in-hospital mortality (*p* < 0.01, Supplementary Figure 4B).

## Discussion

In this multicenter retrospective cohort of patients with sepsis, pre-existing DM was correlated with higher prevalence of stage 1 and stage 3 early SA-AKI, while no meaningful correlation was detected between baseline DM status and in-hospital mortality. Additionally, sustained in-hospital time-weighted mean glucose falling within subgroup-specific intervals was linked to lower odds of early SA-AKI; meanwhile, elevated glycemic values were associated with more severe KDIGO AKI stages and higher in-hospital mortality among patients with stage 1 and stage 2 SA-AKI.

Consistent with ICU and COVID-19 research, pre-existing DM correlates with worse renal injury markers in sepsis patients (4, 13). Related mechanistic studies link this trend to chronic inflammation, endothelial damage and sustained oxidative stress among diabetic individuals (5, 14). However, this retrospective dataset lacks dense serial lab data, so we cannot confirm a one-way causal link from DM to SA-AKI. It is also possible that severe sepsis and early AKI impair insulin sensitivity and trigger hyperglycemia. In this scenario, abnormal glucose levels only reflect critical illness severity instead of acting as a risk factor for renal injury.

Generalized additive models identified distinct glucose intervals that correlated with reduced SA-AKI odds for DM and non-DM subgroups, which may reflect differential metabolic susceptibility to organ injury across patient groups (7, 15). Still, these stratified associative results do not confirm that targeted glycemic management alters renal injury risk. The observed glycemic disparities across subgroups may simply reflect underlying differences in global disease burden rather than representing modifiable causal exposures. Our dataset lacks minute-level paired creatinine and glucose time-series data to fully disentangle temporal order between glycemic derangement and AKI onset, making it impossible to rule out reverse or bidirectional confounding between acute kidney dysfunction and critical illness hyperglycemia.

We also observed that higher time-averaged glucose concentrations correlated with advanced AKI stages and increased in-hospital mortality among mild-to-moderate SA-AKI patients. This alignment with multiple ICU retrospective analyses does not negate the possibility that hyperglycemia serves as a downstream epiphenomenon of overwhelming systemic inflammatory response, instead of a direct mediator of tubular damage (12, 16). Both hyper- and hypoglycemia correlate with disrupted microperfusion and amplified inflammatory cascades, yet we lack longitudinal paired measurements to distinguish whether glycemic fluctuations precede or follow the emergence of renal impairment. Although four independent statistical approaches produced consistent associative patterns to improve result stability, all interpretations remain constrained by the intrinsic limitations of retrospective real-world data. We cannot definitively resolve directional confounding: patients complicated by SA-AKI universally present more severe systemic physiological disturbance, and suboptimal glycemic status may only signal greater illness acuity rather than contribute to kidney injury progression. Further prospective longitudinal research with high-frequency serial creatinine and glucose monitoring is required to delineate precise temporal sequences and untangle bidirectional interactions between glycemic homeostasis and acute renal dysfunction.

It is critical to contextualize our subgroup-specific glucose thresholds against the glucose distribution observed in our cohort. All patients had a time-weighted mean glucose ≥6.1 mmol/L, meaning the 6–11 mmol/L range for diabetic individuals only separates those with persistent hyperglycemia above 11 mmol/L, without capturing hypoglycemic events. Similarly, few non-diabetic patients presented average glucose below 8 mmol/L. Accordingly, the apparent correlation between being “in target” and reduced SA-AKI risk is predominantly the inverse signal of sustained hyperglycemia’s harmful effect on septic kidneys, consistent with prior ICU and COVID-19 cohorts reporting elevated glycemia as a marker of severe organ injury. These asymmetric cutoffs derived from our dataset cannot be generalized to populations with frequent hypoglycemia, and we do not interpret these ranges as balanced dual thresholds to avoid both low and high glucose.

This retrospective observational study included a substantial multicenter cohort of sepsis patients, enabling robust correlative analysis of pre-existing DM and glycemic metrics with early SA-AKI. Unlike prior studies focusing on broad critically ill populations, this work specifically targets early SA-AKI within pathogen-confirmed sepsis patients, delivering subgroup-specific observations of glycemic correlates that add nuance to current ICU literature (2, 3). Multiple adjustment strategies including propensity score matching, inverse probability weighting and doubly robust estimation were applied to mitigate measurable confounding, strengthening the reliability of observed associations. To our knowledge, this multicenter analysis is among the few retrospective studies to report subgroup-specific glycemic intervals correlated with lower SA-AKI odds for diabetic and non-diabetic sepsis patients, which may offer preliminary reference for individualized glycemic monitoring in clinical ICU practice (17–19).

Additionally, the ROC analyses in Supplementary Table 5 demonstrated only marginal discriminative capacity of abnormal glucose levels for predicting early SA-AKI, with AUC values confined between 0.600 and 0.621, merely slightly superior to random chance (AUC = 0.5). This weak predictive performance underscores that dysglycemia cannot act as an independent diagnostic biomarker to identify patients at high risk of early SA-AKI. The onset of SA-AKI is driven by a complex interplay of systemic inflammation, hemodynamic disturbance, pathogen virulence and pre-existing comorbidities, such that isolated time-averaged glucose values lack sufficient discriminatory power when used alone. Our subgroup-specific protective glucose intervals identified via GAM analyses should therefore be interpreted as modifiable risk modifiers for renal injury rather than standalone predictive screening indicators. Clinically, individualized glycemic control targets function as a supportive management strategy to mitigate AKI risk, but cannot replace comprehensive multi-factor risk stratification incorporating infection severity, organ function and hemodynamic parameters. Consistent with prior critical care observational studies, single metabolic indices routinely yield modest AUCs when predicting sepsis-associated organ dysfunction, as no single laboratory variable fully captures the multifactorial pathophysiology of critical illness-induced acute organ injury.

This study has several limitations. First, the retrospective design carries inherent risks of residual unmeasured confounding even after comprehensive multivariable adjustment and propensity balancing. Second, our study population is limited to tertiary hospital ICU patients, so extrapolation to community hospitals and other sepsis populations requires further external validation. Third, while we detected subgroup-specific glycemic ranges correlated with SA-AKI incidence, the exact biological pathways underlying these correlations remain undetermined and require experimental exploration. Fourth, we could not stratify non-diabetic patients into normoglycemia and isolated stress hyperglycemia subgroups, as consistent long-term post-discharge HbA1c records were unavailable in our electronic medical records; prior ICU and COVID-19 studies report divergent renal outcome correlations between transient stress hyperglycemia and chronic DM, which cannot be explored in the present dataset. Fifth, the lack of high-frequency sequential laboratory measurements prevents definitive judgment on temporal order between glycemic dysregulation and AKI onset. SA-AKI itself may exacerbate insulin resistance and glucose disturbance, creating bidirectional confounding that statistical adjustment cannot fully eliminate. All findings reported herein should only be interpreted as observational correlations, and strong causal inferences between glycemic control and SA-AKI risk are unwarranted. Future prospective studies with standardized serial glycemic monitoring and extended metabolic follow-up are necessary to validate our observations and clarify directional relationships.

## Conclusion

This retrospective multicenter study found pre-existing DM correlates with higher early SA-AKI odds, and group-specific in-hospital glucose ranges are linked to lower SA-AKI incidence. Bidirectional interference between AKI and stress hyperglycemia cannot be ruled out in our data, so no causal relationship can be confirmed. Our observational results provide preliminary references for individualized ICU glucose monitoring. Prospective trials and mechanistic research are needed to clarify the interaction between diabetes, stress hyperglycemia and SA-AKI.

## Data Availability

The data analyzed in this study is subject to the following licenses/restrictions: The authors declare that all data in the article are available and can be obtained from the corresponding author upon reasonable request. Requests to access these datasets should be directed to Lina Zhao, 18240198229@163.com.
