# Supplementary figures and images for: Impact of diabetes mellitus and glucose level control on early sepsis-associated acute kidney injury: a multicenter retrospective observational study
Source: Front Med (Lausanne). 2026 Jul 20;13:1878791. doi: 10.3389/fmed.2026.1878791 (PMC13430459; doi:10.3389/fmed.2026.1878791)

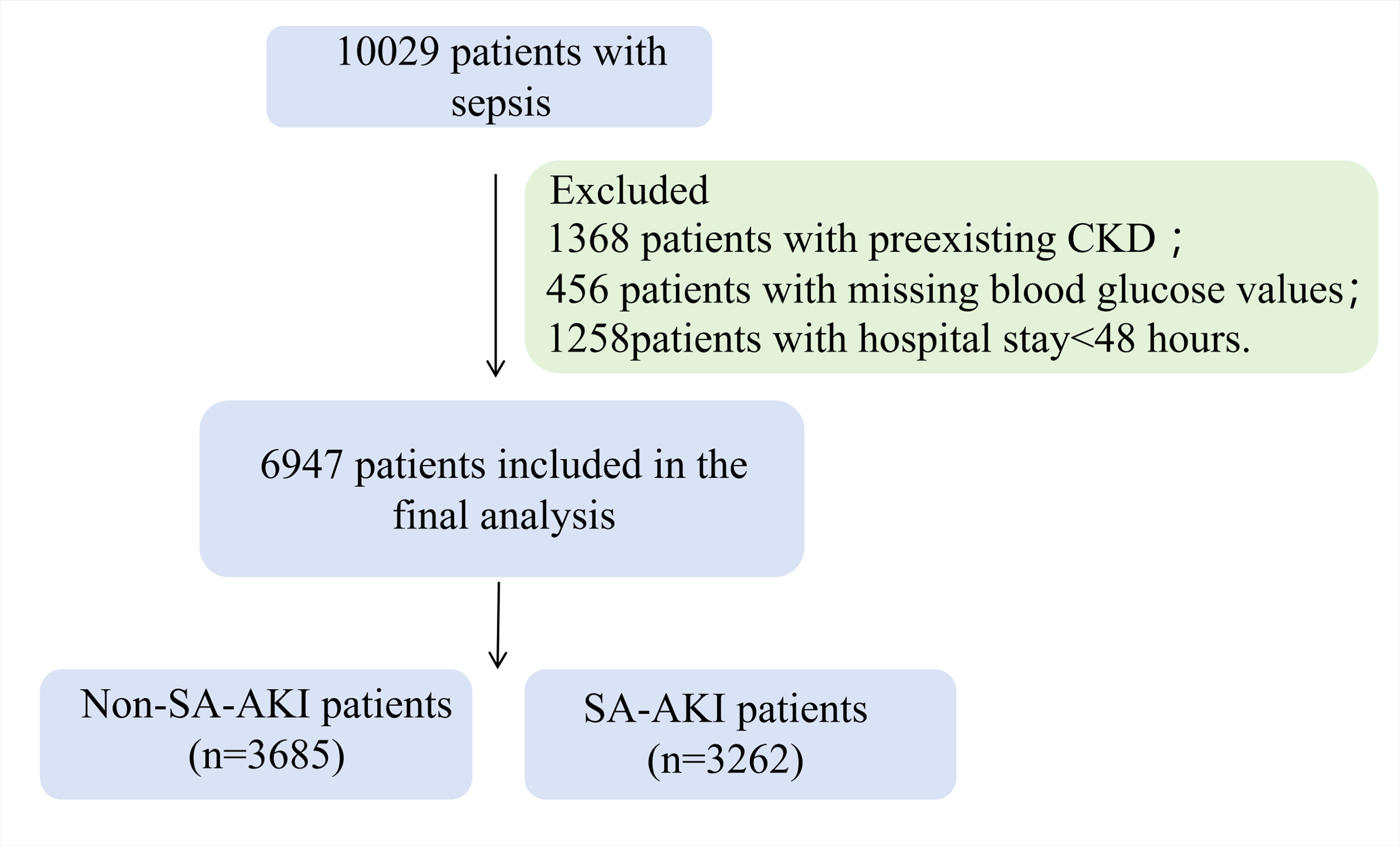

Supplement: Supplementary file 1 [file Image_1.tif]

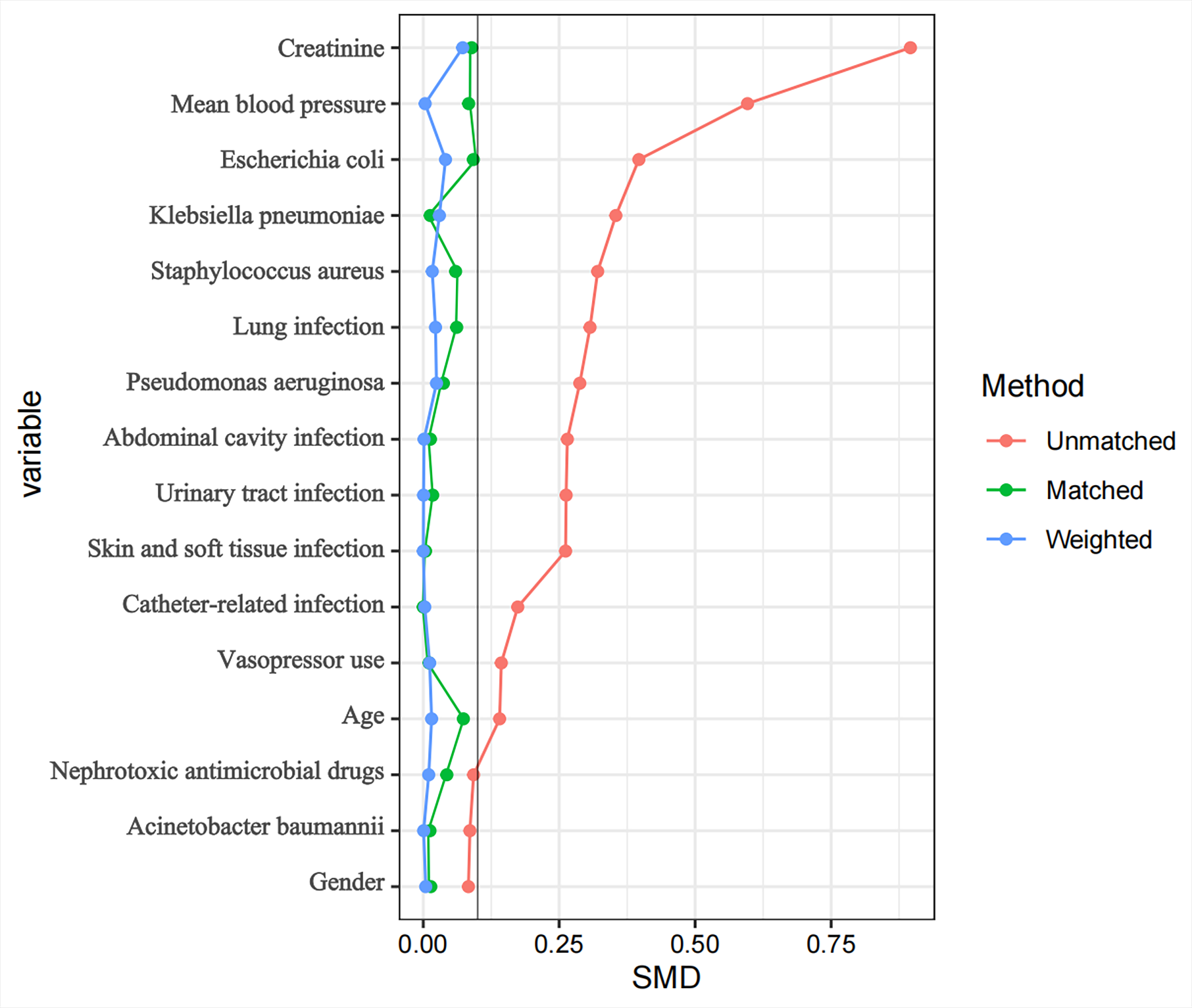

Supplement: Supplementary file 2 [file Image_2.tif]

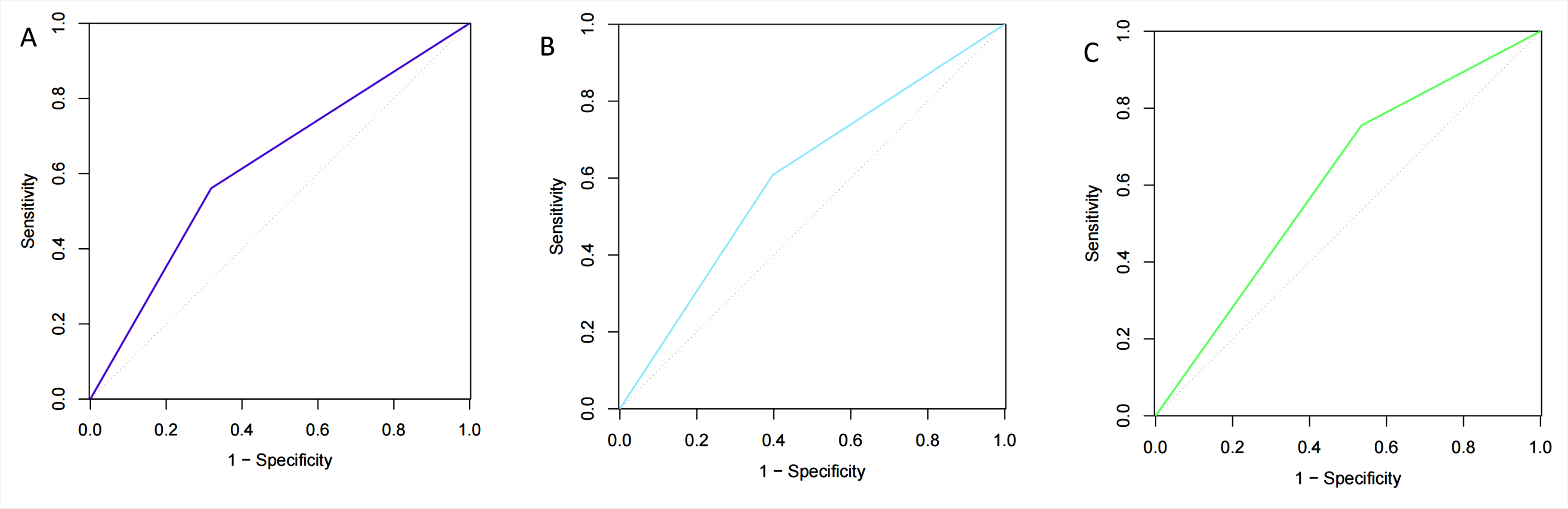

Supplement: Supplementary file 3 [file Image_3.tif]

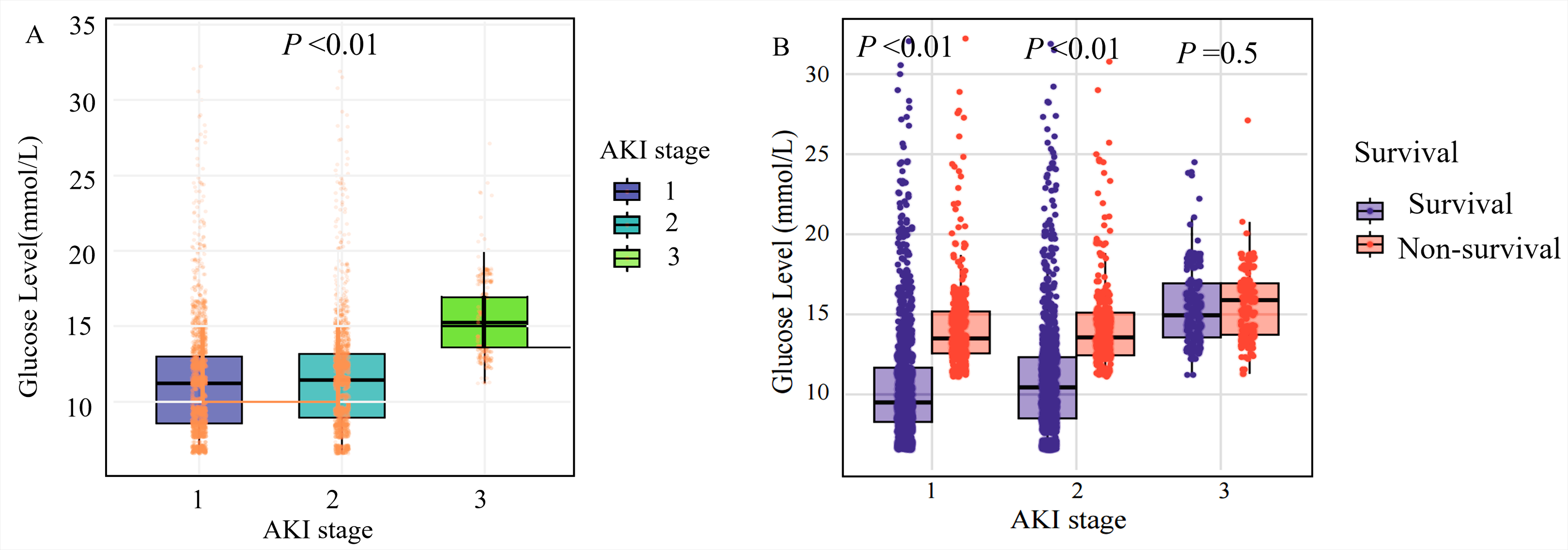

Supplement: Supplementary file 4 [file Image_4.tif]
