# Supplementary material for: Impact of diabetes mellitus and glucose level control on early sepsis-associated acute kidney injury: a multicenter retrospective observational study
Source: Front Med (Lausanne). 2026 Jul 20;13:1878791. doi: 10.3389/fmed.2026.1878791 (PMC13430459; doi:10.3389/fmed.2026.1878791)
Supplement: Supplementary file 5 [file Table_1.docx]

| **Models** | OR | 95% CI | | *P* |
| --- | --- | --- | --- | --- |
|  |  | 2.5% | 97.5% |  |
| **Multivariate Logistic analysis** | | | | |
| Pre-existing diabetes mellitus | 2.54 | 2.25 | 2.86 | <0.001 |
| Blood glucose level 6-11( mmol/L)^*^ | 0.88 | 0.78 | 0.98 | 0.025 |
| Blood glucose level 8-13( mmol/L)^＃^ | 0.55 | 0.48 | 0.62 | <0.001 |
| **Propensity score matching** |  |  |  |  |
| Pre-existing diabetes mellitus | 2.22 | 1.97 | 2.50 | <0.001 |
| Blood glucose level 6-11( mmol/L)^*^ | 0.55 | 0.49 | 0.62 | <0.001 |
| Blood glucose level 8-13( mmol/L)^＃^ | 0.43 | 0.38 | 0.48 | <0.001 |
| **Propensity score** **IPW** | | | | |
| Pre-existing diabetes mellitus | 2.18 | 1.96 | 2.43 | <0.001 |
| Blood glucose level 6-11( mmol/L)^*^ | 0.84 | 0.76 | 0.94 | 0.002 |
| Blood glucose level 8-13( mmol/L)^＃^ | 0.54 | 0.48 | 0.60 | <0.001 |
| **Doubly robust with all covariates** | | | | |
| Pre-existing diabetes mellitus | 1.44 | 1.37 | 1.50 | <0.001 |
| Blood glucose level 6-11( mmol/L)^*^ | 0.94 | 0.89 | 0.98 | <0.001 |
| Blood glucose level 8-13( mmol/L)^＃^ | 0.76 | 0.73 | 0.80 | <0.001 |

**Table 3** Multiple models assessed the relationship between diabetes mellitus and blood glucose levels and the incidence of AKI in sepsis.

IPW: Inverse probability weighting.^*^Patients with pre-existing mellitus;^＃^Patients without pre-existing mellitus; *P* < 0.05 was considered statistically significant.
