# Supplementary material for: Impact of diabetes mellitus and glucose level control on early sepsis-associated acute kidney injury: a multicenter retrospective observational study
Source: Front Med (Lausanne). 2026 Jul 20;13:1878791. doi: 10.3389/fmed.2026.1878791 (PMC13430459; doi:10.3389/fmed.2026.1878791)
Supplement: Supplementary file 6 [file Table_2.docx]

| **eTable 1** **Baseline characteristics of sepsis AKI patients** | | | | | | |
| --- | --- | --- | --- | --- | --- | --- |
|  | Original cohort | | | Match cohort | | |
| **Baseline variables** | Non-SA-AKI patients  (n=3685) | SA-AKI patients  (n=3262) | *P* | Non-SA-AKI patients  (n=2248) | SAKI patients  (n=2248) | *P* |
| **Microbiology type, (n (%))** | | | | | | |
| *Acinetobacter baumannii* | 6(0.2) | 24(0.7) | 0.001 | 6(0.3) | 8(0.4) | 0.789 |
| *Klebsiella pneumoniae* | 119(3.2) | 413(12.7) | <0.001 | 109(4.8) | 131(5.8) | 0.164 |
| *Escherichia Coli* | 275(7.5) | 687(21.1) | <0.001 | 240(10.7) | 224(10.0) | 0.462 |
| *Pseudomonas aeruginosa* | 69(1.9) | 263(8.1) | <0.001 | 63(2.8) | 67(3.0) | 0.789 |
| *Staphylococcus aureus* | 757(20.5) | 1132(34.7) | <0.001 | 593(26.4) | 580(25.8) | 0.684 |
| **Vital signs, (median [IQR])** | | | | | | |
| Heart rate(bpm) | 96.00[88.00,108.00] | 102.00[89.00,119.00] | <0.001 | 96.00[88.00,109.00] | 102.00[89.00,119.00] | <0.001 |
| Respiratory rate (bpm) | 26.00[23.00,30.00] | 28.00[24.00,32.00] | <0.001 | 26.00[23.00,30.00] | 28.00[24.00,33.00] | <0.001 |
| Systolic blood pressure (mmHg) | 86.00[79.00,92.00] | 85.00[76.00,92.00] | <0.001 | 85.00[77.00,91.00] | 86.00[78.00,94.00] | <0.001 |
| Diastolic blood pressure (mmHg) | 46.00[41.00,51.00] | 45.00[39.00,52.00] | <0.001 | 44.00[38.00,48.00] | 46.00[40.00,52.00] | <0.001 |
| Mean arterial pressure(mmHg) | 60.00[55.00,69.00] | 56.00[50.00,62.00] | <0.001 | 57.00[52.00,63.00] | 58.00[52.00,63.00] | 0.331 |
| **Laboratory parameters (median [IQR])** | | | | | | |
| White blood cell (×10^9^ /L) | 14.70[11.40,18.90] | 13.70[9.70,19.20] | <0.001 | 14.65[11.30,19.10] | 13.80[9.90,19.60] | 0.001 |
| Hemoglobin(g/dL) | 9.30[8.20,10.50] | 8.90[7.70,10.40] | <0.001 | 9.25[8.20,10.50] | 9.00[7.80,10.50] | <0.001 |
| Platelet (×10^9^/L) | 134.00[105.00,176.00] | 149.00[103.00,220.00] | <0.001 | 135.00[105.75,181.25] | 147.00[100.75,216.00] | 0.002 |
| Sodium (mmol/l) | 140.00[137.00,141.00] | 140.00[137.00,142.00] | 0.087 | 140.00[138.00,141.00] | 140.00[137.00,142.00] | 0.414 |
| Potassium (mmol/l) | 4.50[4.20,4.80] | 4.60[4.20,5.10] | <0.001 | 4.50[4.20,4.80] | 4.60[4.20,5.20] | <0.001 |
| Lactates (mmol/L) | 1.70[1.20,2.30] | 1.80[1.20,2.70] | <0.001 | 1.70[1.20,2.30] | 1.80[1.30,2.80] | <0.001 |
