# Supplementary material for: Impact of diabetes mellitus and glucose level control on early sepsis-associated acute kidney injury: a multicenter retrospective observational study
Source: Front Med (Lausanne). 2026 Jul 20;13:1878791. doi: 10.3389/fmed.2026.1878791 (PMC13430459; doi:10.3389/fmed.2026.1878791)
Supplement: Supplementary file 7 [file Table_3.docx]

| **eTable 2** Multivariate logistic analysis of risk factors to the incidence in patients with sepsis associated AKI | | | |
| --- | --- | --- | --- |
|  | OR | 95%CI | *P* |
| Age | 1.01 | 1.01-1.02 | <0.001 |
| Gender | 0.85 | 0.75-0.97 | 0.016 |
| **Coexisting illness** | | | |
| Chronic obstructive pulmonary disease | 1.18 | 1.03-1.37 | 0.020 |
| Coronary atherosclerotic heart disease | 1.58 | 1.40-1.78 | <0.001 |
| Diabetes | 2.54 | 2.25-2.86 | <0.001 |
| **Site of infection** | | | |
| Urinary | 0.97 | 0.77-1.23 | 0.809 |
| Lung | 2.26 | 1.74-2.95 | <0.001 |
| Catheter | 0.93 | 0.63-1.39 | 0.729 |
| Skin and soft tissue | 1.77 | 1.29-2.45 | <0.001 |
| Abdominal cavity | 2.13 | 1.54-2.99 | <0.001 |
| **Microbiology type** | | | |
| *Acinetobacter baumannii* | 1.53 | 0.57-4.73 | 0.428 |
| *Klebsiella pneumoniae* | 2.67 | 2.06-3.48 | <0.001 |
| *Escherichia Coli* | 2.24 | 1.85-2.72 | <0.001 |
| *Pseudomonas aeruginosa* | 2.73 | 1.97-3.83 | <0.001 |
| *Staphylococcus aureus* | 1.54 | 1.34-1.76 | <0.001 |
| **Vital signs** |  |  |  |
| Heart rate | 1.01 | 1.01-1.01 | <0.001 |
| Respiratory rate | 1.03 | 1.02-1.04 | <0.001 |
| Systolic blood pressure | 1.08 | 1.07-1.09 | <0.001 |
| Diastolic blood pressure | 1.17 | 1.15-1.19 | <0.001 |
| Mean arterial pressure | 0.80 | 0.78-0.82 | <0.001 |
| **Laboratory parameters** | | | |
| White blood cell | 0.99 | 0.99-1.00 | 0.032 |
| Hemoglobin | 0.95 | 0.92-0.98 | <0.001 |
| Platelet | 1.01 | 1.001-1.002 | <0.001 |
| Potassium | 1.64 | 1.50-1.80 | <0.001 |
| Lactates | 1.19 | 1.15-1.24 | <0.001 |
| **Other index** | | | |
| Use of vasopressors | 0.66 | 0.58-0.75 | <0.001 |
| Mechanical ventilation | 0.45 | 0.38-0.54 | <0.001 |
| Nephrotoxic antimicrobial Drugs | 1.07 | 0.95-1.20 | 0.270 |
