# Supplementary material for: Impact of diabetes mellitus and glucose level control on early sepsis-associated acute kidney injury: a multicenter retrospective observational study
Source: Front Med (Lausanne). 2026 Jul 20;13:1878791. doi: 10.3389/fmed.2026.1878791 (PMC13430459; doi:10.3389/fmed.2026.1878791)
Supplement: Supplementary file 8 [file Table_4.docx]

| **eTable 3** Multivariate logistic analysis of risk factors to the incidence in patients with sepsis and diabetes mellitus associated AKI | | | |
| --- | --- | --- | --- |
|  | OR | 95%CI | *P* |
| Age | 1.01 | 1.01-1.02 | <0.001 |
| Gender | 0.84 | 0.74-0.95 | 0.006 |
| **Coexisting illness** | | | |
| Chronic obstructive pulmonary disease | 1.18 | 1.03-1.36 | 0.020 |
| Coronary atherosclerotic heart disease | 1.6 | 1.42-1.81 | <0.001 |
| **Site of infection** | | | |
| Urinary | 1.05 | 0.84-1.33 | 0.653 |
| Lung | 2.35 | 1.82-3.05 | <0.001 |
| Catheter | 0.98 | 0.67-1.46 | 0.937 |
| Skin and soft tissue | 1.92 | 1.40-2.65 | <0.001 |
| Abdominal cavity | 2.11 | 1.53-2.95 | <0.001 |
| **Microbiology type** | | | |
| *Acinetobacter baumannii* | 1.73 | 0.64-5.27 | 0.300 |
| *Klebsiella pneumoniae* | 2.59 | 2.01-3.36 | <0.001 |
| *Escherichia Coli* | 2.26 | 1.87-2.74 | <0.001 |
| *Pseudomonas aeruginosa* | 2.71 | 1.97-3.78 | <0.001 |
| *Staphylococcus aureus* | 1.62 | 1.42-1.86 | <0.001 |
| **Vital signs** |  |  |  |
| Heart rate | 1.01 | 1.01-1.01 | <0.001 |
| Respiratory rate | 1.03 | 1.02-1.04 | <0.001 |
| Systolic blood pressure | 1.07 | 1.06-1.08 | <0.001 |
| Diastolic blood pressure | 1.15 | 1.13-1.17 | <0.001 |
| Mean arterial pressure | 0.81 | 0.79-0.83 | <0.001 |
| **Laboratory parameters** | | | |
| White blood cell | 0.99 | 0.99-1.00 | 0.006 |
| Hemoglobin | 0.95 | 0.92-0.98 | 0.001 |
| Platelet | 1.01 | 1.001-1.001 | <0.001 |
| Potassium | 1.64 | 1.50-1.79 | <0.001 |
| Lactate | 1.20 | 1.15-1.24 | <0.001 |
| Glucose | 0.88 | 0.78-0.98 | 0.025 |
| **Other index** |  |  |  |
| Use of vasopressors | 0.65 | 0.57-0.74 | <0.001 |
| Mechanical ventilation | 0.46 | 0.39-0.54 | <0.001 |
| Nephrotoxic antimicrobial Drugs | 1.20 | 1.07-1.34 | 0.002 |
