# Supplementary material for: Impact of diabetes mellitus and glucose level control on early sepsis-associated acute kidney injury: a multicenter retrospective observational study
Source: Front Med (Lausanne). 2026 Jul 20;13:1878791. doi: 10.3389/fmed.2026.1878791 (PMC13430459; doi:10.3389/fmed.2026.1878791)
Supplement: Supplementary file 9 [file Table_5.docx]

| **eTable 4** Multivariate logistic analysis of risk factors to the incidence in patients with sepsis without diabetes mellitus associated AKI | | | |
| --- | --- | --- | --- |
|  | OR | 95%CI | *P* |
| Age | 1.01 | 1.01-1.02 | <0.001 |
| Gender | 0.83 | 0.73-0.94 | 0.004 |
| **Coexisting illness** | | | |
| Chronic obstructive pulmonary disease | 1.17 | 1.01-1.34 | 0.032 |
| Coronary atherosclerotic heart disease | 1.58 | 1.41-1.79 | <0.001 |
| **Site of infection** | | | |
| Urinary | 1.06 | 0.84-1.34 | 0.625 |
| Lung | 2.35 | 1.82-3.05 | <0.001 |
| Catheter | 0.93 | 0.63-1.38 | 0.722 |
| Skin and soft tissue | 1.94 | 1.42-2.69 | <0.001 |
| Abdominal cavity | 2.17 | 1.57-3.03 | <0.001 |
| **Microbiology type** | | | |
| *Acinetobacter baumannii* | 1.6 | 0.60-4.85 | 0.369 |
| *Klebsiella pneumoniae* | 2.49 | 1.93-3.24 | <0.001 |
| *Escherichia Coli* | 2.28 | 1.89-2.77 | <0.001 |
| *Pseudomonas aeruginosa* | 2.75 | 1.99-3.84 | <0.001 |
| *Staphylococcus aureus* | 1.65 | 1.44-1.89 | <0.001 |
| **Vital signs** |  |  |  |
| Heart rate | 1.01 | 1.01-1.01 | <0.001 |
| Respiratory rate | 1.03 | 1.02-1.04 | <0.001 |
| Systolic blood pressure | 1.07 | 1.06-1.08 | <0.001 |
| Diastolic blood pressure | 1.15 | 1.14-1.17 | <0.001 |
| Mean arterial pressure | 0.81 | 0.79-0.82 | <0.001 |
| **Laboratory parameters** | | | |
| White blood cell | 0.99 | 0.99-1.00 | 0.004 |
| Hemoglobin | 0.94 | 0.91-0.97 | <0.001 |
| Platelet | 1.01 | 1.001-1.002 | <0.001 |
| Potassium | 1.62 | 1.48-1.77 | <0.001 |
| Lactates | 1.19 | 1.14-1.23 | <0.001 |
| Glucose | 0.55 | 0.48-0.62 | <0.001 |
| **Other index** |  |  |  |
| Use of vasopressors | 0.66 | 0.58-0.75 | <0.001 |
| Mechanical ventilation | 0.45 | 0.38-0.54 | <0.001 |
| Nephrotoxic antimicrobial Drugs | 1.19 | 1.06-1.33 | 0.004 |
